# Regulatory Components of the Alternative Complement Pathway in Endothelial Cell Cytoplasm, Factor H and Factor I, Are Not Packaged in Weibel-Palade Bodies

**DOI:** 10.1371/journal.pone.0121994

**Published:** 2015-03-24

**Authors:** Nancy A. Turner, Sarah E. Sartain, Shiu-Ki Hui, Joel L. Moake

**Affiliations:** 1 Department of Bioengineering, Rice University, Houston, Texas, United States of America; 2 Department of Pediatrics, Section of Hematology-Oncology, Texas Children’s Cancer and Hematology Centers, Houston, Texas, United States of America; 3 Baylor College of Medicine, Houston, Texas, United States of America; 4 Division of Transfusion Medicine and Coagulation, Texas Children’s Hospital, Houston, Texas, United States of America; National Cerebral and Cardiovascular Center, JAPAN

## Abstract

It was recently reported that factor H, a regulatory component of the alternative complement pathway, is stored with von Willebrand factor (VWF) in the Weibel-Palade bodies of endothelial cells. If this were to be the case, it would have therapeutic importance for patients with the atypical hemolytic-uremic syndrome that can be caused either by a heterozygous defect in the factor H gene or by the presence of an autoantibody against factor H. The in vivo Weibel-Palade body secretagogue, des-amino-D-arginine vasopressin (DDAVP), would be expected to increase transiently the circulating factor H levels, in addition to increasing the circulating levels of VWF. We describe experiments demonstrating that factor H is released from endothelial cell cytoplasm without a secondary storage site. These experiments showed that factor H is not stored with VWF in endothelial cell Weibel-Palade bodies, and is not secreted in response in vitro in response to the Weibel-Palade body secretagogue, histamine. Furthermore, the in vivo Weibel-Palade body secretagogue, DDAVP does not increase the circulating factor H levels concomitantly with DDAVP-induced increased VWF. Factor I, a regulatory component of the alternative complement pathway that is functionally related to factor H, is also located in endothelial cell cytoplasm, and is also not present in endothelial cell Weibel-Palade bodies. Our data demonstrate that the factor H and factor I regulatory proteins of the alternative complement pathway are not stored in Weibel-Palade bodies. DDAVP induces the secretion into human plasma of VWF —- but not factor H.

## Introduction

Von Willebrand factor (VWF) is a large multimeric protein composed of monomeric subunits (~250 kDa) linked by disulfide bonds into large polymers. VWF multimers are synthesized in human vascular endothelial cells (ECs) and in megakaryocytes. [1] The multimers are stored in the Weible-Palade bodies (WPBs) of endothelial cells, as well as in the alpha granules of megakaryocytes and their derivative circulating cells, platelets. [2] (WPBs and alpha granules are analogous organelles with similar contents.) When stimulated, ECs secrete ultra-large (UL), hyper-adhesive VWF multimers that initiate platelet adhesion. [3,4] The ULVWF multimers are secreted rapidly from EC WPBs in response to a variety of agonists primarily through mechanisms resulting in either increases in internal calcium (i.e. histamine/H1 receptors) or cAMP levels (i.e. vasopressin/V2 receptors). [5,6] Under basal conditions, ULVWF is secreted at a low rate from EC WPBs. [7]

We recently demonstrated that human umbilical vein endothelial cells (HUVECs) express the mRNA for complement components, and synthesize and release the associated proteins. [8] These proteins include the components of the alternative complement pathway, including the negative complement regulatory proteins, factor (F) H and FI. (FH and FI are functionally related control proteins.) In our initial investigation of the release of complement components from HUVECs, we did not detect organelle storage of any complement protein, including FH and FI, prior to release from HUVECs. [8] A more recent article made the contradictory contention that FH is stored in HUVEC WPBs and is co-secreted with VWF during cell stimulation. [9] Our additional optical and functional data presented in this paper indicate that FH and FI are not present with VWF in WPBs, and that FH is not secreted along with VWF in vivo in response to DDAVP.

We have further investigated by fluorescent image analysis and by measurement of FH release from stimulated and un-stimulated HUVECs whether or not FH is stored in HUVEC WPBs in vitro, or released continuously from HUVEC cytoplasm. Furthermore, we have measured the in vivo release of FH into the plasma of patients given the WPB secretagogue, des-amino-D-arginine vasopressin (DDAVP), during clinical evaluation of von Willebrand disease (VWD). We have, furthermore, conducted additional image analysis to determine the precise endothelial cell location of FI.

## Methods

### Ethics Statement

The Rice Institutional Review Board (IRB) approved all of the experiments on human endothelial cells in this study. Blood and tissue samples were collected under a protocol approved by the Rice IRB and donors provided their written informed consent to participate in the study. Protocol Name: Processing of Large von Willebrand Factor (VWF) Multimers: VWF Cleavage, Thrombosis and Platelet Aggregation, Protocol Number: 11-183E. The Rice IRB reviews protocols annually and has approved of this consent procedure and study through 5/13/2015.

Plasma samples from pediatric patients with type 1 VWD were collected and tested under a protocol approved by the Baylor College of Medicine IRB. Donors provided their written informed consent to participate in the study. Protocol Name: Correlation of Clinical Phenotype and Laboratory Parameters in Children with Von Willebrand Disease, Protocol Number: H-29626, Expiration date: 8/19/2015.

### Human umbilical vein endothelial cells (HUVECs)

Primary HUVECs were isolated from human umbilical veins. Umbilical cords were washed with phosphate buffer (140 mM NaCl, 0.4 mM KCl, 1.3 mM NaH_2_PO_4_, 1.0 mM Na_2_HPO_4_, 0.2% glucose, pH 7.4) and then infused with a collagenase solution (0.02% in PBS) for 30 min at room temperature. The cords were rinsed with 100 ml of phosphate buffer, and eluates containing endothelial cells were centrifuged at 250g for 10 min. The cell pellets were re-suspended and further maintained in MCDB basal medium (M131, Sigma-Aldrich) with additions of low-serum growth supplement plus penicillin, streptomycin and glutamine (PSG, Life Technologies). In experiments for sample collection, HUVECs were maintained in serum-free medium consisting of M131 with PSG plus insulin, transferrin and selenium (ITS, Life Technologies).

### Immunofluorescent Microscopy

HUVECs grown on gelatin-coated glass coverslips were washed with PBS, fixed with 1% p-formaldehyde in PBS, and then treated with 0.02% Triton-X to allow internal staining. The cells were stained for 15 min with the primary and fluorescent antibody pairs described below. The cell nuclei were detected with 1.5 μg/ml 4’,6-diamidino-2-phenylindole (DAPI) included in the mounting medium (Fluoro-Gel II, Electron Microscopy Sciences). Fluorescent images were acquired using IP Lab software version 3.9.4r4 with a fluorescence colocalization module (Scanalytics, Inc., Fairfax, VA) on a Nikon Diaphot TE300 microscope equipped with CFI Plan Fluor 60X oil, numerical aperture 1.4 and CFI Plan Apo Lambda 100X oil, numerical aperture 1.45 objectives plus 10X projection lens (Nikon, Garden City, NY), SensiCamQE CCD camera (Cooke Corp., Romulus, MI), motorized stage and dual filter wheels (Prior) with single band excitation and emission filters for FITC/TRITC/CY5/DAPI (Chroma, Rockingham, VT). Image areas acquired at 60X are 78 μm x 58 μm and image areas acquired at 100X are 41 μm x 30 μm.

### Internal HUVEC FH, VWF, β-actin and FI Detection

The following primary and fluorescently labeled secondary antibody pairs were used to detect internal FH, VWF, β-actin and FI in HUVECs:

#### FH and VWF Antibody Pair 1

10 μg/ml polyclonal goat anti-human FH [Complement Technologies, Tyler, TX; catalogue number (cat. #) A237] plus secondary antibody chicken anti-goat Alexa Fluor (AF) IgG-647 (cat. # A21469); and 10 μg/ml polyclonal rabbit anti-human VWF (Ramco Laboratories, Sugarland, TX) plus secondary antibody donkey anti-rabbit AF IgG-488 (cat. # A21441). The antigen used to generate the Ramco polyclonal rabbit anti-human VWF antibody was VWF purified in our laboratory from human cryoprecipitate purchased at the Gulf Coast Regional Blood Center (Houston, TX).

#### FH and VWF Antibody Pair 2

Two mouse monoclonal antibodies to human FH at 10 μg/ml each (Quidel cat. # A229 and Thermo Fisher Scientific/Pierce clone L20/3) plus goat anti-mouse AF F(ab’)_2_ fragment-IgG-647 (cat. # A21237); and 10 μg/ml polyclonal rabbit anti-human VWF plus secondary fluorescent donkey anti-rabbit antibody AF IgG-488.

#### FH and β-actin Antibody Pair 3

Two mouse monoclonal antibodies to human FH at 10 μg/ml each (Quidel cat. # A229 and Thermo Fisher Scientific/Pierce clone L20/3) plus secondary antibody donkey anti-mouse AF IgG-488 (cat. # A21202); and 2 μg/ml polyclonal goat anti-β-actin (I-19, Santa Cruz Biotechnology, cat. # sc-1616) plus secondary antibody chicken anti-goat AF IgG-647.

#### FH and FI Antibody Pair 4

Two mouse monoclonal antibodies to human FH at 10 μg/ml each (Quidel cat. # A229 and Thermo Fisher Scientific/Pierce clone L20/3) plus secondary antibody chicken anti-mouse AF IgG-647 (cat. # A21463); and 10 μg/ml polyclonal goat anti-human factor I (Complement Technologies, cat. # A238) plus secondary antibody donkey anti-goat AF IgG-488 (cat. # A21202).

The AF-labeled secondary antibodies were used at final concentrations of 20 μg/ml and were purchased from Life Technologies. The polyclonal antibodies made against human FH and FI have previously been shown to be monospecific in detecting their target proteins in human serum and in FH- and FI-depleted serum by Western blot techniques. [8]

### Fluorescence Colocalization Measurements

Proteins within HUVECs were detected in two channels using primary antibodies combined with fluorescently labeled secondary antibodies. The data were analyzed for similarity in shape, location and intensity. FH was compared for degree of colocalization with VWF, β-actin, and with FI. The antibodies in Pair 2 were used in analyses comparing FH and VWF and the antibodies in Pair 4 were used to compare FH and FI. In both comparisons, VWF and FI were detected in the green (488 nm) channel (probe 1) and FH was detected in the red (647 nm) channel (probe 2). For colocalization comparison of FH and β-actin, the antibodies in Pair 3 were used; FH was detected in the green channel (probe 1) and β-actin was detected in the red channel (probe 2).

Prior to analysis, the images at 60X and 100X had non-subjective background subtraction. Fluorescence cross-talk and bleed-through were controlled by choosing fluorochromes on secondary detection antibodies with distinct, non-overlapping spectra and narrow bandwidth filters. [10] The acquisition of images without any cross-talk or bleed through was confirmed experimentally. VWF in HUVEC WPBs was antibody-labeled with AF-488 or AF-647 on separate slides and images were acquired in both channels.

The extent of similarity between two channels was determined using intensity scatter plots and software calculated values of Pearson’s correlation coefficient (PCC) and Manders’ coefficients (M1 and M2). [10–13] The PCC ranges from −1 to 1, where negative values show an inverse relationship between the two probes. Zero indicates no correlation and 1 indicates complete correlation between the two channels. The PCC provides shape correlation by comparing the intensity distribution of the two channels. PCC values are independent of high background levels. In most cases, scatter plots of green channel intensity (probe 1) verses red channel intensity (probe 2) from a merged image of 2 spatially correlated proteins should show a single linear relationship between the two channels. A merged image with perfect overlap distribution of the red and green channels at equal intensities will result in a centered line (where y = x) and a Pearson’s coefficient value of 1.

Manders’ coefficients, M1 and M2, range from 0 to 1, and these values represent the portion of the intensity in each channel that overlaps with intensity in the other channel (1 and 2 refer to probe 1 and probe 2). These values are not dependent on intensity differences between probe 1 and 2, but require background-subtracted images. [10–12] Because PCC and M1 and M2 calculate signal overlap in different ways, a single positive measured value (0.5 or higher) indicates colocalization. [11,13]

### Factor H and VWF Protein Measurements

#### FH Fluorescent Immunoassay

Black 96-well plates were coated with 200 ng/well of polyclonal goat anti-human FH (Complement Technologies, cat. # A237) in 100 mM bicarbonate buffer, pH 9.6, overnight (ON) at 4°C. TBS-T washed wells were blocked ON with 1% immunoglobin-free BSA in PBS (BSA/PBS), followed by ON incubation with test samples or purified FH protein (Complement Technologies) for the standard curve (with a range of 3.9–250 ng/ml). Each type of sample was diluted in BSA/PBS. TBS-T washed wells were next incubated with 170 ng/ml of mouse monoclonal antibody to human FH (Thermo Fisher Scientific/Pierce clone L20/3) for 30 min followed by incubation with 0.5 μg/ml of secondary goat anti-mouse IgG-HRP (Rockland Immunochemicals, Limerick, PA). Fluorescence was measured in a Tecan Infinite M200 Pro plate reader after the addition of the HRP substrate ADHP (10-Acetyl-3, 7-dihydroxyphenoxazine; AnaSpec, Fremont, CA) with excitation at 560 nm and emission at 590 nm.

The high range of sensitivity of the FH immunoassay is based on ADHP, a substrate for HRP that reacts with hydrogen peroxide to produce a highly fluorescent product. The raw fluorescent readings for the FH standards (3.9 ng/ml to 250 ng/ml) range from 1000 to 40,000. Reciprocal plots of FH standard dilutions (1/conc.) versus fluorescence intensity at 590 nm (1/590 Intensity) produce linear equations that allow the interpolation of FH concentrations from 0 to 3.9 ng/ml in addition to values between 3.9 and 250 ng/ml (S1 Fig.).

#### VWF Fluorescent Immunoassay

VWF levels were measured in HUVEC supernatant samples using a protocol similar to the FH immunoassay. The black 96-well plates were coated at 100 ng/well with polyclonal rabbit anti-human VWF (Ramco Laboratories). The standard curve was constructed using normal human pooled plasma diluted in BSA/PBS. The VWF was detected using 1 μg/ml polyclonal goat anti-human VWF (Bethyl Laboratories, cat. # A80-138) plus 0.5 μg/ml rabbit anti-goat IgG-HRP (Life Technologies). Fluorescence was measured 30 min after the addition of the ADHP substrate with excitation at 560 nm and emission at 590 nm in the Tecan plate reader.

#### Factor H and VWF Levels in HUVECs

FH and VWF antigen levels were measured by fluorescent immunoassays in culture supernatant samples collected from 100 μM histamine-stimulated or un-stimulated HUVECs into serum-free medium. Samples were collected at 30 min and at 1, 3, 5 and 7 hours after cell stimulation or addition of fresh medium (to unstimulated cells) and stored at −20°C until assayed.

#### Patient Factor H and VWF Measurements

FH and VWF antigen levels were measured in citrated plasma samples obtained from 6 pediatric patients with type 1 VWD undergoing a protocol evaluation for circulating VWF response to DDAVP at Texas Children’s Hospital. Five of 6 patients were given Stimate, the nasal preparation of DDAVP (150 μg or 300 μg, depending on weight). One patient was given Desmopressin, the intravenous formulation of DDAVP (0.3 μg/kg).

VWF antigen was measured by standard clinical laboratory techniques on the day of collection. FH antigen levels were measured by fluorescent immunoassay in samples stored at −80°C up to 3 months. For each patient, VWF and FH antigens were measured in samples obtained prior to DDAVP administration, and in samples obtained 1 and 4 hours later. The Baylor College of Medicine IRB approved the additional FH measurements on patient plasma samples.

#### Normal donor FH measurements

Six single donor units of plasma anticoagulated with citrate-phosphate-dextrose were obtained from the Gulf Coast Regional Blood Center (Houston, TX) and stored at 4°C for 26 days. Aliquots were removed at days 1, 14 and 26 after storage for comparison of FH levels by fluorescent immunoassay. The IRB at Rice University approved the purchase and use of human blood products.

## Results

### Internal HUVEC FH and VWF Detection

**Fig 1:**
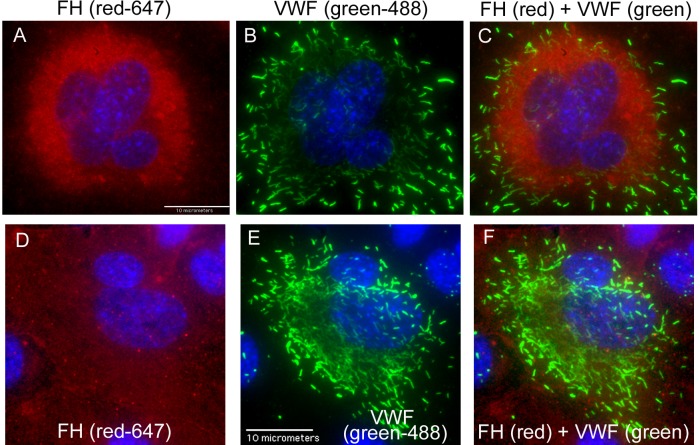
Factor H is not present in HUVEC WPBs. Un-stimulated HUVECs were fixed with 1% p-formaldehyde and treated with 0.02% Triton-X to allow intracellular staining, and then stained with rabbit anti-human VWF antibody plus chicken anti-rabbit IgG-Alexa Fluor (AF) 488-secondary antibody. In panels A, B and C, VWF staining was followed by staining with two mouse anti-human FH monoclonal antibodies plus goat anti-mouse F(ab’)_2_-IgG-AF 647-secondary antibody. Cells were imaged at 100X and cell nuclei were detected with DAPI (blue). (A) FH detection, red-647 channel; (B) VWF detection, green-488 channel and (C) merged image with simultaneous VWF and FH detection. In panels D, E and F, VWF staining was followed by FH detection using goat anti-human FH antibody plus donkey anti-goat IgG-AF 647-secondary antibody and imaging at 60X. (D) FH detection, red-647 channel; (E) VWF detection, green-488 channel; and (F) merged image with simultaneous VWF and FH detection. Images shown are representative of VWF and FH staining in >10 separate HUVEC cultures.

Using immunofluorescent microscopy and non-overlapping spectral secondary antibody pairs, FH was detected throughout the HUVEC cytoplasm (Fig. 1A and 1D), whereas only VWF was detected in HUVEC WPBs (Fig. 1B and 1E). The cytoplasmic location of FH in HUVECs was confirmed by separate detection of FH using both monoclonal antibodies and monospecific polyclonal antibodies directed against human FH. The superimposed images of VWF and FH detection, using either the polyclonal or monoclonal antibodies, did not show structural or fluorescent overlap (Fig. 1C and 1F). These results are strong evidence that VWF and FH do not co-localize in HUVEC WPBs.

### Fluorescence Colocalization Measurements

#### Colocalization measurements of FH and VWF

The separate cellular location of FH from VWF within HUVECs observed in the fluorescent images was verified by calculations of Pearson’s (PCC) and Manders’ (M1 and M2) colocalization correlation coefficients in the merged images. The PCC measurement describes the number of locations within the merged image with similar red and green intensity levels. [11] In the merged images of FH and VWF, the average PCC value was low (0.13 and 0.17) for images acquired at 60X and 100X, respectively (Table 1 and S1 Dataset). These values signify low correlation between the locations of FH and VWF.

**Table 1:** Colocalization coefficients of internal FH in HUVECs concurrently stained with VWF, β-actin and FI.

	**FH and VWF, 60X Mean ± SD (range)**	**FH and β-Actin, 60X Mean ± SD (range)**	**FH and FI, 60X Mean ± SD (range)**
**PCC**	0.17 ± 0.10 (0.1–0.3)	0.43 ± 0.06 (0.4–0.5)	0. 66^a^ ± 0.15 (0.47–0.90)
**M1**	0.30 ± 0.08 (0.2–0.5)	0.77^a^ ± 0.03 (0.7–0.8)	0.82^a^ ± 0.06 (0.73–0.93)
**M2**	0.36 ± 0.09 (0.2–0.5)	0.80^a^ ± 0.03 (0.8–0.9)	0.84^a^ ± 0.06 (0.75–0.96)
	**FH and VWF, 100X Mean ± SD (range)**	**FH and β-Actin, 100X Mean ± SD (range)**	**FH and FI, 100X Mean ± SD (range)**
**PCC**	0.13 ± 0.1 (0.1–0.3)	0.39 ± 0.08 (0.3–0.5)	0.64^a^ ± 0.17 (0.36–0.87)
**M1**	0.22 ± 0.09 (0.1–0.4)	0.86^a^ ± 0.05 (0.8–0.9)	0.84^a^ ± 0.10 (0.66–0.98)
**M2**	0.28 ± 0.14 (0.1–0.5)	0.90^a^ ± 0.04 (0.8–0.97)	0.87^a^ ± 0.09 (0.70–1.00)

Colocalization coefficients, Pearson’s (PCC) and Manders’ (M1 and M2), were measured in HUVEC images stained concurrently for FH and with VWF, β-actin, or FI, using the antibody pairs 2, 3 and 4 described under Internal HUVEC FH, VWF, β-actin and FI Detection in the Methods section. Data were analyzed in 2–4 images per experiment from 4–7 experiments with each antibody pair.

^a^Indicates signal correlation in the two channels.

**Fig 2:**
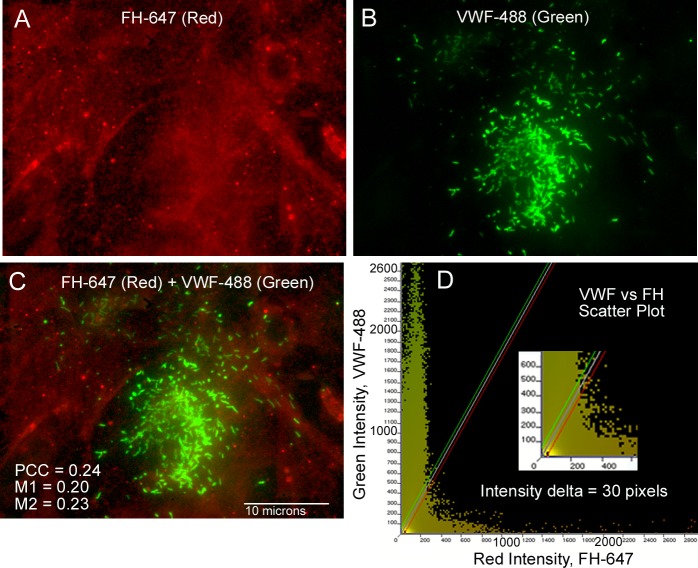
Intensity scatter plots comparing FH with VWF do not show colocalization or a single correlation. HUVECs were treated and stained for FH (red) and VWF (green) as detailed in Fig. 1AB. Images are shown without the DAPI-stained nuclei. (A) FH detection with mouse anti-FH + goat anti-mouse AF-647 (red); (B) VWF detection with rabbit anti-VWF + chicken anti-rabbit AF-488 (green); and (C) Merged image of (A) and (B) detecting FH and VWF. The correlation coefficients, Pearson’s (PCC) and Manders’ (M1 and M2), for these 2 images are on the left bottom of the image. (D) The intensity scatter plot of the merged image in (C) showing 2 linear independent relationships of the data. The scattering of points along each of the axes indicates a low degree of signal overlap. The intensity delta is the distance from either outer color line to the plot’s center of density line and defines the area considered for colocalization. An intensity delta of 30 pixels was used for all of the colocalization measurements. The inset in (C) shows an enlargement of the plot’s origin.

Fig. 2 shows representative images of FH (Fig. 2A), VWF (Fig. 2B), the merged image (Fig. 2C), and the resulting intensity scatter plot of the merged image (Fig. 2D). The scatter plot has 2 distinct populations, parallel to each of the axes showing low correlation between the two signals. The population along the x-axis shows that the distribution of intensity values for FH in the red (647 nm) channel are only in locations where very low intensity values were detected for VWF in the green (488 nm) channel. The reverse situation describes the vertical population along the y-axis; i.e., the green VWF intensity distribution was only in locations with very low red intensity levels.

Similar results were obtained using the Manders’ correlation coefficients, M1 and M2, to evaluate the extent of FH and VWF colocalization. M1 and M2 express the fraction of each channel probe that overlaps with the other channel probe, independent of signal intensity. [11] The values of M1 and M2 calculated from merged images of FH and VWF indicated that < 1/3 of the green VWF signal overlapped with the red FH signal and ~1/3 of the red FH signal had overlapping locations with the green VWF signal (Table 1, Fig. 2C and S1 Dataset). Only merged images with values for Manders’ coefficients between 0.5 and 1 are considered to have colocalized signals. [13] Only a single positive measured value (0.5 or higher) for PCC or M1 and M2 is necessary to show a degree of colocalization. In the FH and VWF merged images, neither the PCC value nor the values calculated for M1 and M2 were above 0.5 and, therefore, the values confirm that these 2 proteins occupy separate locations within HUVECs.

#### Colocalization measurements of FH with β-actin and FI

**Fig 3:**
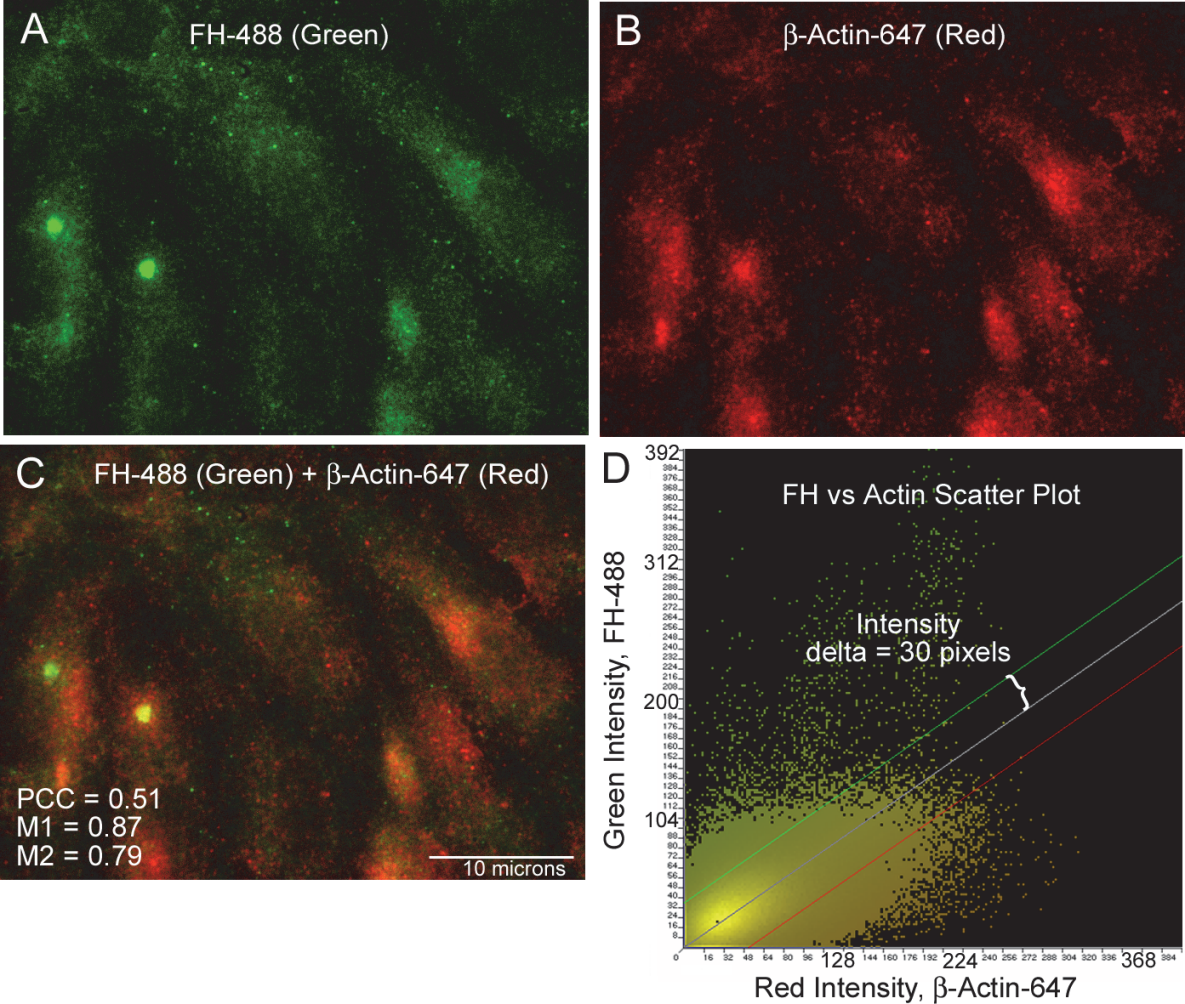
Intensity scatter plots of FH with β-actin shows a colocalization in the HUVEC cytoplasm. HUVECs were formaldehyde-fixed, treated with Triton-X and stained concurrently for FH and **β**-actin. (A) FH was detected using 2 mouse monoclonal antibodies to human FH plus donkey anti-mouse AF IgG-488 (green). (B) **β**-actin was detected using polyclonal goat anti- **β**-actin plus chicken anti-goat antibody AF IgG-647 (red). (C) Shows the merged image detecting both FH (green) and **β**-actin (red) and the calculated values for the Pearson’s (PCC) and Manders’ (M1 and M2) correlation coefficients. (D) The intensity scatter plot of the merged image in (C) shows a single linear correlation indicative of a signal overlap.

The interior HUVEC location of FH was compared with the cytoplasmic structural protein, **β**-actin, using the same colocalization techniques and calculations described above. The comparison of FH and **β**-actin indicated that these two proteins share common cellular locations (Fig. 3). The intensity scatter plot from the representative FH and **β**-actin merged image demonstrates a single linear relationship between the two channels (Fig. 3D). The calculated correlation coefficients from FH and **β**-actin merged images support the fact that FH and **β**-actin are both located in the HUVEC cytoplasm (S1 Dataset). Although the PCC levels were below 0.5 (0.43 at 60X and 0.39 at 100X), the M1 and M2 values (between 0.77 and 0.9) indicate a high degree of colocalization (Table 1). As stated above, either a positive (>0.5) Pearson’s or Manders’ correlation coefficient value is sufficient to indicate some colocalization.

**Fig 4:**
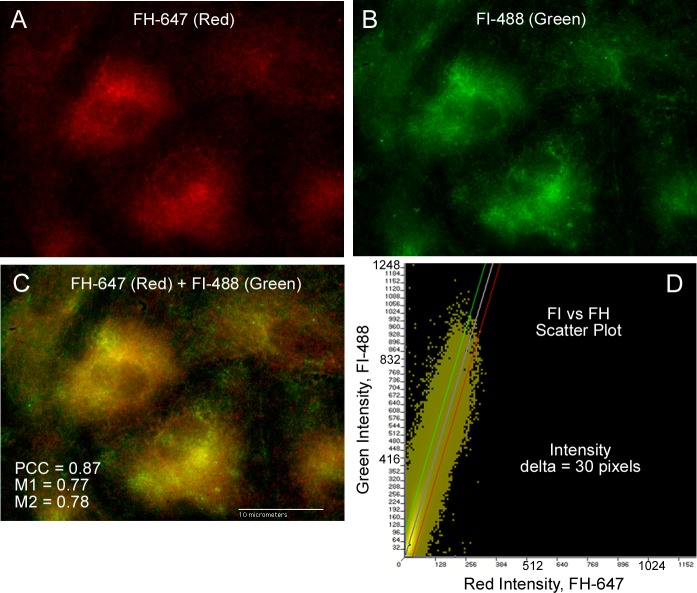
Intensity scatter plots of FH with FI indicate a high degree of colocalization in the HUVEC cytoplasm. HUVECs were formaldehyde-fixed, treated with Triton-X and stained concurrently for FH and FI. (A) FH was detected using 2 mouse monoclonal antibodies to human FH plus chicken anti-mouse AF IgG-647 (red). (B) FI was detected using polyclonal goat anti-human FI plus donkey anti-goat AF IgG-488 (green). (C) Shows the merged image detecting both FH (red) and FI (green) and the calculated values for the Pearson’s (PCC) and Manders’ (M1 and M2) correlation coefficients. (D) The intensity scatter plot of the merged image in (C) shows a single linear correlation indicative of a signal overlap. The population is skewed towards the y-axis on account of the higher green intensity values of FI detection.

Table 1 also includes the colocalization correlation coefficients obtained from analysis of merged image of FH and FI. Values of both colocalization coefficients were positive for signal correlation also indicating a shared cellular cytoplasmic location for these two complement regulatory proteins. The PCC values calculated from merged images of FH and FI (0.66 at 60X and 0.64 at 100X) were ~4-fold higher than PCC values for FH and VWF, and moderately higher than PCC values of FH and **β**-actin. The M1 and M2 values for FH and FI averaged between 0.82 and 0.87, indicating a detection overlap (S1 Dataset). Fig. 4 shows representative images of FH (Fig. 4A), FI (Fig. 4B), the merged image (Fig. 4C), and the merged image intensity scatter plot (Fig. 4D).

### Factor H and VWF Protein Measurements

#### FH and VWF secretion from HUVECs

**Fig 5:**
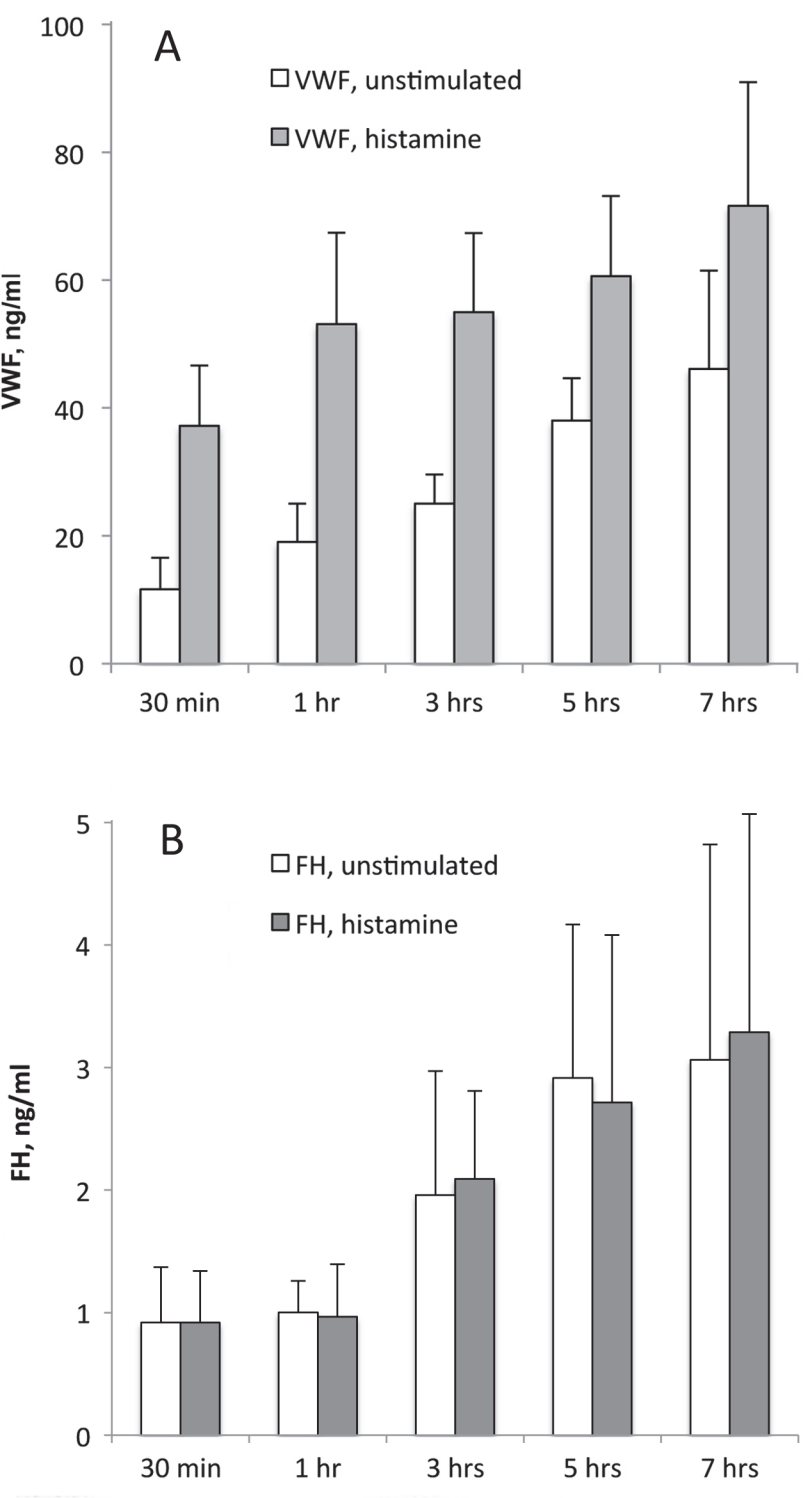
FH release from HUVECs is not increased with histamine stimulation. Cell supernatants collected from 30 min to 7 hours after 100μM histamine stimulation or from unstimulated HUVECs in serum-free medium were quantified for *(*A) VWF levels and (B) FH levels by fluorescent immunoassays. Values shown (ng/ml) are means plus standard deviations from samples collected from 4–6 separate experiments. In (A) VWF levels were significantly different at each time point (P < 0.001 or 0.01) in samples from un-stimulated HUVECs compared to histamine-stimulated HUVECs.

Additional evidence that FH is not located in WPBs or other HUVEC storage vesicle was obtained by In vitro measurements of VWF and FH released from un-stimulated and stimulated HUVECs. Histamine stimulation resulted in ~4-fold increases in VWF concentrations at 30 min and 1 hour (increases of 25 and 34 ng/ml), and 2-fold increases at 3 hours (30 ng/ml increase), compared to levels measured in un-stimulated HUVECs at the same time points (Fig. 5A and S2 Dataset). In contrast to VWF, FH levels were similar in all HUVEC supernatants regardless of histamine stimulation at each time point measured, *i*.*e*., there was no difference in FH levels between histamine stimulated and un-stimulated HUVECs (Fig. 5B and S3 Dataset). This continuous release of FH, along with the absence of FH secretion in response to histamine-stimulation, indicates that FH is not stored in HUVEC WPBs with contents (e.g., VWF) destined for stimulated secretion.

#### FH measurements in stored plasma

FH is a structurally stable protein and when purified from plasma and retains its functional activity under a wide range of temperatures and pH conditions. [14] In our in vivo studies, FH antigen was measured in patient citrated plasma samples that were collected and stored at −80°C for up to 3 months. In parallel studies, we measured the FH levels in 6 individual units of plasma anticoagulated with citrate-phosphate-dextrose and stored at 4°C for 26 days. The FH levels measured on the first day of storage in these plasma samples compared to the FH levels measured after 14 and 26 days of storage were not significantly changed (Table 2 and S4 Dataset), indicating that our measurement of FH antigen in stored patient plasma samples were not likely to be influenced by storage (at −80°C).

**Table 2:** Changes in FH levels measured in plasma stored at 4°C for 26 days.

Unit#	Day 1 FH, μg/ml	Day 14 FH, μg/ml	Day 26 FH, μg/ml
**1**	364	391	387
**2**	445	400	404
**3**	423	363	372
**4**	461	519	467
**5**	471	432	454
**6**	530	508	552

Six individual units of plasma anticoagulated with citrate-phosphate-dextrose were stored at 4°C for 26 days. The FH levels were measured by fluorescent immunoassay on day 1 and after 14 and 26 days of storage at 4°C.

#### FH and VWF levels in patient plasma after DDAVP

**Fig 6:**
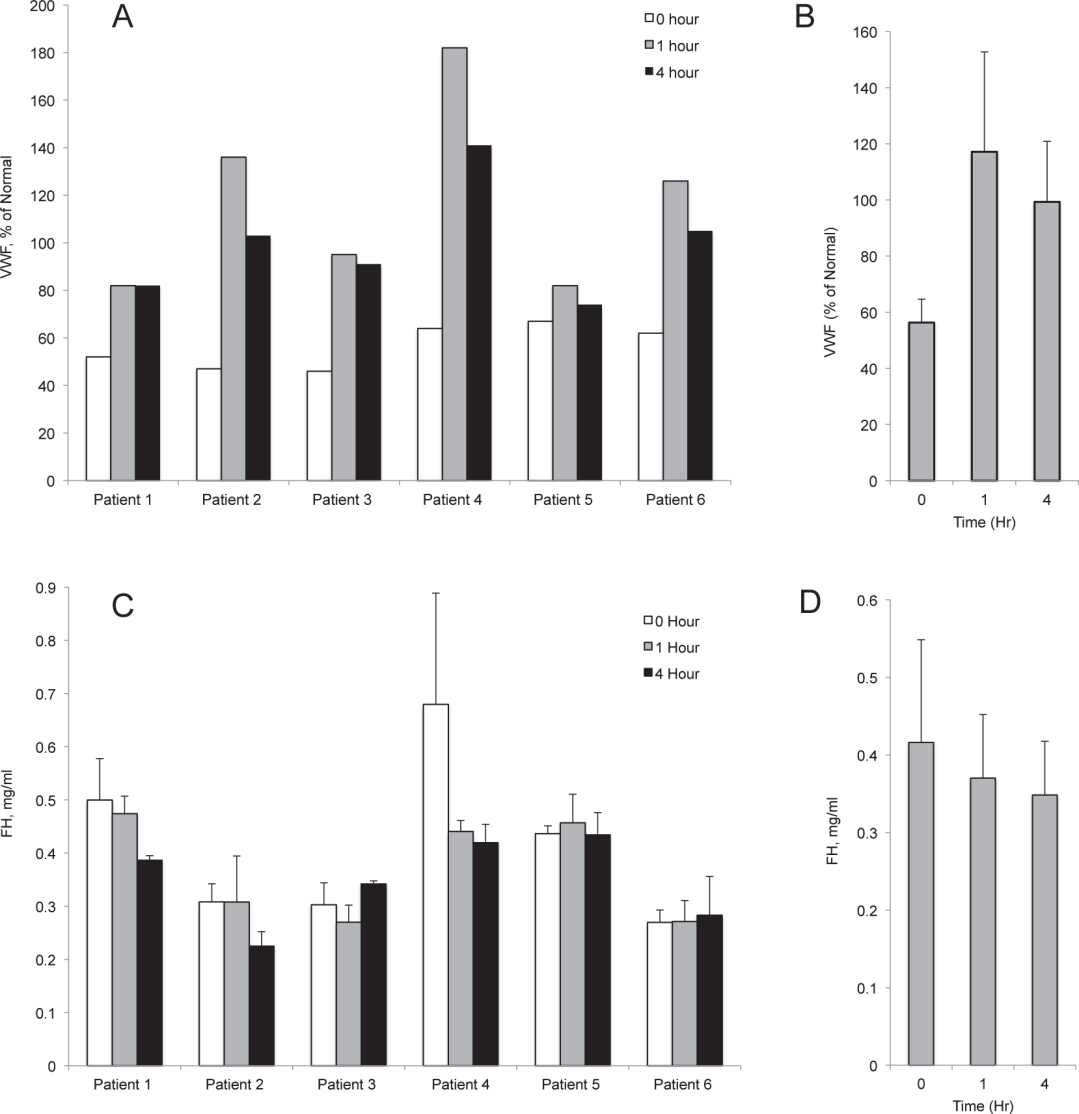
VWF antigen levels increase in VWD patients after DDAVP administration without increases in FH levels. VWF and FH antigen levels were measured in citrated plasma samples from 6 VWD pediatric patients treated with DDAVP. VWF antigen levels were measured by standard clinical laboratory techniques and FH levels were determined by fluorescent immunoassays. For each patient, VWF and FH levels were measured in samples obtained prior to DDAVP administration, and in samples obtained 1 and 4 hours later. (A) Individual VWF levels in plasma samples. Values are in percentage of normal plasma VWF level. (B) The average VWF plasma levels of the 6 patients at the 3 different times, plus standard deviations. The mean response of VWF antigen to DDAVP was significantly greater 1-hour and 4-hours post-DDAVP compared with baseline (P = 0.01 and 0.007, respectively). (C) Individual FH levels of the same 6 patients before and after DDAVP administration. The values shown are means plus standard deviations from 3 FH immunoassays. There was either no change, or a decrease in plasma FH concentrations, at 1-hour and 4-hours post-DDAVP. (D) The average FH plasma level of the 6 patients at the 3 different time points. There was no statistically significant difference in the mean responses of FH to DDAVP among the 3 time points (P = 0.3 for baseline to 1-hour and P = 0.2 for baseline to 4-hours).

In all 6 VWD pediatric patients studied, VWF antigen levels increased in plasma samples at 1-hour post-DDAVP compared to baseline, and remained elevated 4-hours later (Fig. 6A and S5 Dataset). The mean difference in plasma VWF in response to DDAVP was significantly greater in both 1-hour and 4-hours post-DDAVP samples compared to baseline values (P values of 0.01 and 0.007, respectively) (Fig. 6B and S5 Dataset). In contrast, in the same 6 patient samples, there was no increase in the individual plasma FH levels in samples at 1-hour or 4-hours post-DDAVP administration, compared to pre-DDAVP plasma concentrations (Fig. 6C and S6 Dataset). When combined, there was a slight decrease in the average FH plasma level of the 6 patients at 1-hour or 4-hours post-DDAVP (Fig. 6D and S6 Dataset). (P = 0.3 for baseline to 1-hour and P = 0.2 for baseline to 4-hours).

## Discussion

In a previous report investigating alternative complement convertase formation in HUVECs, we observed that FH (and other alternative complement proteins) were in the cytoplasm of HUVECs. [8] It has also been demonstrated by other investigators that FH was not detected in the α-granules of platelets (the organelle analogue of EC WPBs) using either fluorescent confocal microscopy or electron microscopy. [15] In contrast, in early 2014, Rayes, et al reported FH and VWF colocalization in HUVEC WPBs by using two secondary antibodies with wavelengths that have some spectral overlap (488 nm for FH and 555 nm for VWF). [9] In the report by Rayes, et al, the high intensity of VWF fluorescence in WPBs (detected at 555 nm) may have caused a “bleed through” into the 488 nm channel and caused the authors to identify mistakenly this latter fluorescence as “FH” co-localizing with VWF in WPBs. (Many published articles contain guidelines for fluorescent image acquisition and subcellular colocalization analysis.) [10,11,13,16] In our current experiments, we used non-overlapping spectral secondary detection antibody pairs (488 nm for VWF and 647 nm for FH), and did not detect FH in HUVEC WPBs.

Specifically, the conclusion drawn by Rayes, et al [9] that FH is present along with VWF in HUVEC WPBs was based on two types of experiments done by their group: detection of FH and VWF in HUVEC WPBs using fluorescent microscopy; and measurement of VWF and FH levels released into HUVEC supernatant with and without heme exposure. In their fluorescent microscopy experiments, FH was detected using 3 biotinylated monoclonal antibodies against FH plus secondary streptavidin-FITC (excitation peak at 495 and emission peak at 519). VWF was detected with a polyclonal rabbit antibody against VWF plus secondary goat anti-rabbit IgG-Alexa Fluor (AF)-555 (excitation 555 and emission 565). The emission spectrum for FITC ranges from 486 to 656 nm and the emission spectrum range for AF-555 is 540 to 705 nm. There is a spectral overlap from 540 nm through 655 nm that would allow the VWF-AF-555 emission signal to be detected in the FH-FITC (488 nm) channel. Because FH is located throughout the HUVEC cytoplasm, the single-channel images of FH detection shown by Rayes, et al show a green cytoplasmic background plus green WPBs from the AF-555 signal “bleed through”. Their merged images of FH and VWF have green FH cytoplasmic staining plus yellow WPBs indicating overlapping detection in both channels. In their paper, the authors did not question why FH could have 2 separate cellular locations (cytoplasmic and WPBs). The Method section in the Rayes, et al article did not provide details about instrumentation, image acquisition, and number of experiments. [9]

In contrast, in this current manuscript we detected VWF using rabbit anti-human VWF antibody plus chicken anti-rabbit IgG-AF-488-secondary antibody, and detected FH with either a combination of two mouse anti-human FH monoclonal antibodies [one of these (Quidel A229) was also used in the Rayes, et al study [9]] plus goat anti-mouse F(ab’)_2_-IgG-AF 647-secondary antibody or with a polyclonal goat anti-human FH antibody plus donkey anti-goat IgG-AF 647-secondary antibody. The emission spectrum for AF-488 is the same as for FITC 9described in the proceeding paragraph). The excitation peak for AF-647 is 650 and the emission peak is at 668 nm. The emission spectral range for AF-647 is from 629 to 800 nm, and there are no overlapping spectral regions for AF-647 and AF-488. Furthermore, our previous 2013 PLoS ONE article contains a figure (Figure 11) demonstrating the lack of signal “bleed-through” in our fluorescent microscope system. [8] In these previously published experiments, VWF was detected with AF-488 in HUVEC WPBs and cytoplasmic complement proteins were detected with AF-594-labeled secondary antibodies. We measured the signal intensities along lines dissecting WPBs in the 488-nm channel and intensities at identical locations detected in the 594-nm channel in order to prove the lack of signal “bleed-through”.

In the Rayes, et al article [9], levels of FH and VWF were measured in untreated HUVEC supernatant (control) or HUVECs exposed to 100 **μ**M heme for 30 min. Their VWF and FH levels increased from ~35 to 80 ng/ml and from ~0.5 to 1.7 ng/ml, respectively. The levels were measured by an unspecified immunoassay at a single time point (30 min), and the number of experiments was not stated. Exposure to heme has previously been shown to cause oxidative stress and apoptosis in vascular endothelial cells. [17–19] The increased levels of FH and VWF in the heme exposed HUVECs could have been the result of cell injury. In contrast, in our experiments in this current manuscript, FH levels were measured in 4–6 separate HUVEC cultures in samples collected after 30 min, 1, 3, 5, and 7 hours under histamine-stimulating and non-stimulating conditions. (Histamine is an established HUVEC WPB secretagogue.) [20–23] The FH levels in samples collected at the 30 min and 1-hour time points (each data point is an average of 2 determinations, a total of 20 data points) range from 0.48 to 1.8 ng/ml (S4 Dataset).

Colocalization correlation coefficients were calculated in our study in order to support mathematically our conclusions from fluorescent images that FH is not located with VWF in HUVEC WPBs. The Pearson’s coefficient was used to determine the linear relationship between the intensity measurements in the two channels, whereas the Manders’ coefficients (M1 and M2) were used to calculate the fractional overlap of each signal in the other channel. [10,11] These correlation values are frequently used to determine whether or not two proteins coexist in the same cellular location. [11] Our data were obtained in images without spectral overlap and, for M1 and M2, were processed with background subtraction.

DDAVP is effective therapy for type 1 VWD because the agent stimulates the secretion of WPB contents and increases circulating VWF. [6,24] Both our in vitro and in vivo experiments demonstrated that, in contrast to VWF, neither of the complement regulatory proteins, FH and FI, is packaged in, or secreted from, the WPBs of histamine-stimulated human ECs. FH and FI are released from HUVECs after synthesis and processing without an intermediate storage site. In addition, DDAVP-induced VWF secretion is not accompanied by FH secretion in individuals exposed to the agent.

## Supporting Information

S1 Dataset Colocalization coefficients of internal FH in HUVECs concurrently stained with VWF, β-actin and FI. Colocalization coefficients, Pearson’s (PCC) and Manders’ (M1 and M2), were measured in HUVEC images stained concurrently for FH and with VWF, β-actin, or FI.
(XLSX)

S2 Dataset VWF levels measured in HUVEC supernatants with and without histamine stimulation. Cell supernatants collected from 30 min to 7 hours after 100μM histamine stimulation or from unstimulated HUVECs in serum-free medium were quantified for VWF levels and by fluorescent immunoassays.
(XLSX)

S3 Dataset FH levels measured in HUVEC supernatants with and without histamine stimulation. Cell supernatants collected from 30 min to 7 hours after 100μM histamine stimulation or from unstimulated HUVECs in serum-free medium were quantified for FH levels and by fluorescent immunoassays.
(XLSX)

S4 Dataset Changes in FH levels measured in plasma stored at 4°C for 26 days. Six individual units of plasma anticoagulated with citrate-phosphate-dextrose were stored at 4°C for 26 days. The FH levels were measured by fluorescent immunoassay on day 1 and after 14 and 26 days of storage at 4°C.
(XLSX)

S5 Dataset VWF antigen levels measured in VWD patients after DDAVP administration. VWF antigen levels were measured in citrated plasma samples from 6 VWD pediatric patients treated with DDAVP. VWF antigen levels were measured by standard clinical laboratory techniques.
(XLSX)

S6 Dataset FH antigen levels measured in VWD patients after DDAVP administration. FH antigen levels were measured in citrated plasma samples from 6 VWD pediatric patients treated with DDAVP. FH levels were determined by fluorescent immunoassays.
(XLSX)

S1 Fig Reciprocal plots of FH protein standards to show the lower limits of the FH immunoassay. The high range of sensitivity of the FH immunoassay is βased on ADHP (10-Acetyl-3, 7-dihydroxyphenoxazine), a substrate for HRP that reacts with hydrogen peroxide to produce a highly fluorescent product with excitation at 530 nm and emission at 590 nm. The raw fluorescent intensities for the FH standards (3.9 ng/ml to 250 ng/ml) range from 1000 to 40,000 (Table I in S1 Fig.). Shown are 3 reciprocal plots of FH standard dilutions (1/conc.) versus fluorescence intensity at 590 nm (1/590 Intensity). Plot 1 shows the full range of FH standards (3.9 ng/ml to 250 ng/ml), Plot 2 shows the 4 lowest FH concentrations (3.9 ng/ml to 31.25 ng/ml) and Plot 3 shows the 4 lowest FH concentrations plus the y-intercept point. The linear relationship allows for the interpolation of FH concentrations between 0 and 3.9 ng/ml.
(PDF)
