# Supplementary material for: Regulatory Components of the Alternative Complement Pathway in Endothelial Cell Cytoplasm, Factor H and Factor I, Are Not Packaged in Weibel-Palade Bodies
Source: PLoS One. 2015 Mar 24;10(3):e0121994. doi: 10.1371/journal.pone.0121994 (PMC4372330; doi:10.1371/journal.pone.0121994)

Table S1

	FH ng/ml	Intensity 1	Intensity 2	Mean	-BSA	1/intensity
A	BSA	1247	1128	1187.5	0	
B	3.90625	6799	7003	6901	5713.5	0.00018
C	7.8125	11744	11902	11823	10635.5	9.4E-05
D	15.625	18415	18916	18665.5	17478	5.7E-05
E	31.25	27063	27830	27446.5	26259	3.8E-05
F	62.5	34994	35090	35042	33854.5	3E-05
G	125	37525	40422	38973.5	37786	2.6E-05
H	250	39165	41260	40212.5	39025	2.6E-05

FH ng/ml	1/conc	1/intensity
3.90625	0.256	0.000175
7.8125	0.128	9.4E-05
15.625	0.064	5.72E-05
31.25	0.032	3.81E-05
62.5	0.016	2.95E-05
125	0.008	2.65E-05
250	0.004	2.56E-05

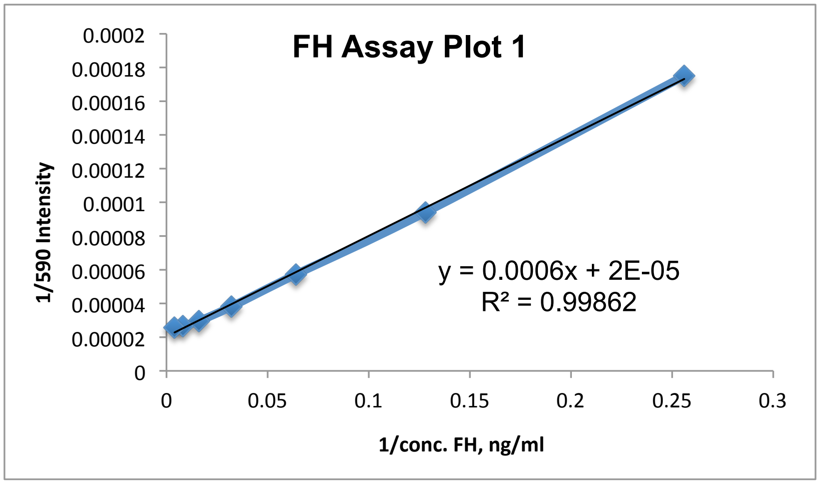

FH ng/ml	1/conc	1/intensity
3.90625	0.256	0.000175
7.8125	0.128	9.4E-05
15.625	0.064	5.72E-05
31.25	0.032	3.81E-05

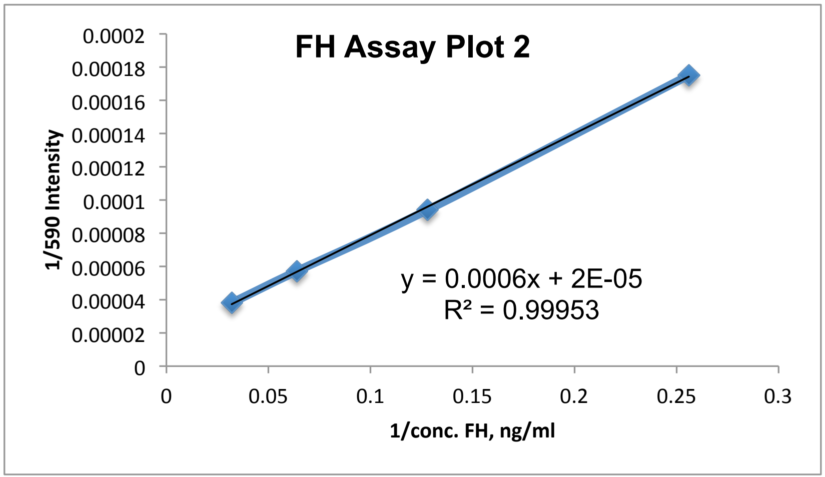

FH ng/ml	1/conc	1/intensity
3.90625	0.256	0.000175
7.8125	0.128	9.4E-05
15.625	0.064	5.72E-05
31.25	0.032	3.81E-05
0	0	2.00E-05

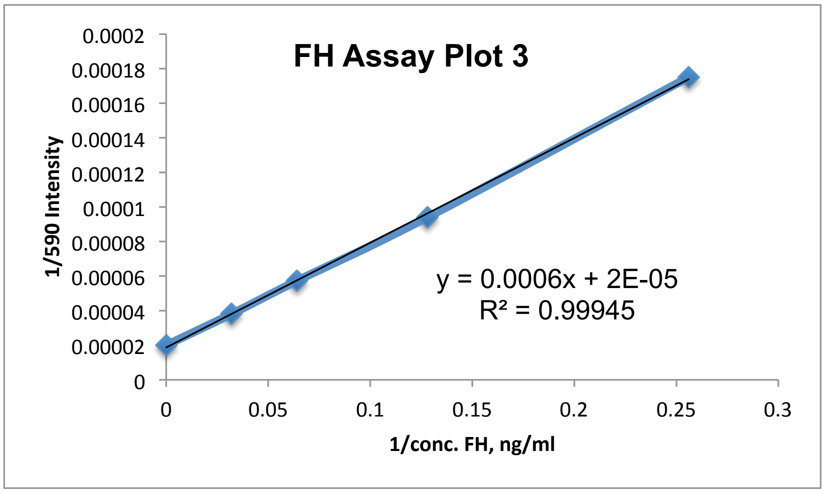

Supplement: S1 Fig — The high range of sensitivity of the FH immunoassay is βased on ADHP (10-Acetyl-3, 7-dihydroxyphenoxazine), a substrate for HRP that reacts with hydrogen peroxide to produce a highly fluorescent product with excitation at 530 nm and emission at 590 nm. The raw fluorescent intensities for the FH standards (3.9 ng/ml to 250 ng/ml) range from 1000 to 40,000 (Table I in S1 Fig.). Shown are 3 reciprocal plots of FH standard dilutions (1/conc.) versus fluorescence intensity at 590 nm (1/590 Intensity). Plot 1 shows the full range of FH standards (3.9 ng/ml to 250 ng/ml), Plot 2 shows the 4 lowest FH concentrations (3.9 ng/ml to 31.25 ng/ml) and Plot 3 shows the 4 lowest FH concentrations plus the y-intercept point. The linear relationship allows for the interpolation of FH concentrations between 0 and 3.9 ng/ml. (PDF) [file pone.0121994.s007.pdf]
